# Two Cultivars of Peanut (*Arachis hypogaea*) Show Different Responses to Iron Deficiency

**DOI:** 10.3390/cimb48010099

**Published:** 2026-01-18

**Authors:** Lei Chen, Zifei Liu, Lei Zhou, Hong Wang

**Affiliations:** 1 Heilongjiang Academy of Black Soil Conservation and Utilization, No. 368, Xuefu Road, Harbin 150086, China; chengz5251@163.com; 2 State Key Laboratory of Efficient Utilization of Arid and Semi-Arid Arable Land in Northern China, Institute of Agricultural Resources and Regional Planning, Chinese Academy of Agricultural Sciences, Beijing 100081, China; 3 Heilongjiang Academy of Sciences, No. 204, Zhongshan Road, Harbin 150001, China; 4 China National Center for Quality Inspection and Test of Chemical Fertilizers (Beijing), Chinese Academy of Agricultural Sciences, Beijing 100081, China

**Keywords:** ferric chelate reductase, iron deficiency, IRON-REGULATED TRANSPORTER1, peanut, photosynthetic parameter

## Abstract

**Background**: Peanut is susceptible to iron (Fe) deficiency, particularly in calcareous soils. However, comparative studies on the adaptive mechanisms of different peanut cultivars to Fe deficiency remain limited. This study aimed to investigate the physiological and molecular responses of two distinct peanut cultivars to Fe deprivation and to identify the key traits contributing to differential Fe efficiency. **Methods**: Two peanut cultivars, LH11 and YZ9102, were cultivated under Fe-sufficient and Fe-deficient conditions, using both hydroponic and pot-based soil culture systems. Multiple parameters were assessed, including visual symptomology, biomass, tissue Fe concentration, active Fe in leaves, chlorophyll (Chl) content (SPAD value), net photosynthetic rate (Pn), Chl fluorescence (Fv/Fm), rhizosphere pH, root ferric chelate reductase (FCR) activity, and the relative expression of two Fe-acquisition-related genes (*AhIRT1* and *AhFRO1*) via qRT-PCR. **Results**: Cultivar YZ9102 exhibited more severe Fe deficiency chlorosis symptoms, which also appeared earlier than in LH11, under both cultivation systems. Under Fe deficiency, YZ9102 showed significantly lower Chl content, Pn, and Fv/Fm compared to LH11. In contrast, LH11 demonstrated a greater capacity for rhizosphere acidification and maintained significantly higher root FCR activity under Fe-limited conditions. Gene expression analysis revealed that Fe deficiency induced the up-regulation of *AhIRT1* and *AhFRO1* in the roots of LH11, while their transcript levels were suppressed or unchanged in YZ9102. **Conclusions**: The peanut cultivar LH11 possesses superior tolerance to Fe deficiency compared to YZ9102. This enhanced tolerance is attributed to a synergistic combination of traits: the maintenance of photosynthetic performance, efficient rhizosphere acidification, heightened root Fe^3+^ reduction capacity, and the positive transcriptional regulation of key Fe uptake genes. These findings provide crucial insights for the selection and breeding of Fe-efficient peanut varieties for cultivation in Fe-deficient environments.

## 1. Introduction

Peanut (*Arachis hypogaea* L.) is a vital global oilseed and food legume, with China being the world’s leading producer, contributing 33.6% of the world’s output [1], largely from the calcareous soils of the Northern China Plain. Domestically, peanuts account for about 30% of the nation’s total oilseed production [2]. However, productivity in these regions is severely constrained by Fe deficiency chlorosis, a pervasive agronomic challenge. In calcareous soils, high pH and elevated bicarbonate levels drastically reduce the solubility and bioavailability of Fe, rendering it largely inaccessible to plants [2,3]. While Fe fertilization is a common corrective measure, the development and use of Fe-efficient cultivars offers a more sustainable and economically viable long-term strategy [4].

Fe ranks as the fourth most abundant element in the Earth’s crust. In soils, it primarily occurs in two forms: ferrous (Fe^2+^) and ferric (Fe^3+^). However, it becomes unavailable for plants grown in aerated alkaline soils in which Fe hydroxides, oxyhydroxides, and oxides with low solubility are easily produced under high pH and high bicarbonate content [2,5].

As an essential micronutrient, Fe plays a critical role in numerous physiological processes in plants, including photosynthesis, respiration, and nitrogen fixation [6]. Fe serves as an crucial component of electron chains and a co-factor in numerous enzymes, most notably within iron–sulfur clusters and heme proteins. It is also required for the biosynthesis of Chl in plants [6]. Deficiency in Fe rapidly inhibits Chl production, leading to the characteristic symptom of interveinal chlorosis in young leaves [7]. At the physiological level, Fe deficiency disrupts the antenna system of photosystem II (PSII), resulting in a lower proportion of open reaction centers and reduced PSII efficiency [8,9,10]. Moreover, it adversely affects the structure, development, and function of the entire photosynthetic apparatus [11], culminating in a significant decline in Pn [6,12]. However, while indispensable for plant metabolism, excess Fe can be detrimental. It catalyzes the production and accumulation of reactive oxygen species and free radicals (H_2_O_2_, ^•^OH, O_2_^•−^, HO_2_), which cause oxidative damage to cellular components, such as lipids, proteins, and DNA [13,14].

To cope with Fe deficiency, plants have developed sophisticated mechanisms to mobilize and acquire Fe from soil [15,16,17,18]. Grasses employ Strategy II, which is characterized by enhancing the release of phytosiderophores (PSs) [19] and the subsequent uptake of the Fe(III)-PS complex via specific transporters of the YSL family [19]. In contrast, dicots and non-grass monocots utilize Strategy I. Their responses to Fe deficiency include the proliferation of root-hairs, development of transfer cells, acidification of the rhizosphere, increases in FCR activity, and up-regulation of Fe(II) transporters [15]. Rhizosphere acidification is primarily mediated by AHA2, a H^+^-translocating P-type ATPase, which promotes the solubilization of the chelated Fe(III) complex in soils [20]. Fe(III) is reduced to Fe(II) by a plasma membrane-bound FCR before being taken up by root cells [15]. FCRs belong to the ferric reductase oxidase (FRO) family. The first gene encoding a root-expressed FCR identified in *Arabidopsis* was *FERRIC-REDUCTION OXIDASE2* (*FRO2*) [21]. The resulting Fe(II) is transported across the root epidermis via the high-affinity Fe(II) transporter IRON-REGULATED TRANSPORTER1 (IRT1) [22,23]. IRT1, the first identified member of the ZRT/IRT-like protein (ZIP) family localized in the plasma membrane [24], also transports other divalent cations such as Cd^2+^ and Zn^2+^ [25,26]. Under Fe-limited conditions, the expression of several basic helix–loop–helix (*bHLH*) transcription factors, such as *Fer-like Iron Deficiency-Induced Transcription Factor* (*FIT*), *Popeye* (*PYE*), and *PYE homolog IAA-Leu Resistant-3* (*ILR3*), is up-regulated in *Arabidopsis* [16,27,28]. Other genes such as *YABBY* [29], *FE UPTAKE-INDUCING PEPTIDE1* (*FEP1*) or *IRON MAN3* (*IMA3*) [30], *MANNOSE-BINDING-LECTIN* 1 (*MNB1*) [31], and *bHLH1b* [6] may also contribute to enhancing Fe availability and maintaining Fe homeostasis in plants [16].

This study aims to systematically investigate the physiological and molecular basis of differential Fe deficiency tolerance in the contrasting peanut cultivars LH11 and YZ9102. Using integrated hydroponic and soil-based systems, we will evaluate a suite of traits including growth, photosynthetic performance, root physiological responses (FCR activity, proton extrusion), and the expression of key Fe-acquisition genes. By linking root-level acquisition strategies with whole-plant physiological performance, this work seeks to identify the key traits that constitute Fe efficiency in peanut. The findings are expected to provide crucial insights for the strategic breeding of Fe-efficient peanut varieties and for optimizing sustainable management practices in Fe-limiting calcareous soils.

## 2. Materials and Methods

### 2.1. Experiment 1: Physiological and Molecular Responses of Hydroponically Grown Peanut to Fe Supply

#### 2.1.1. Hydroponically-Grown Peanut and Treatment

The seeds of two peanut cultivars, ‘LH11’ and ‘YZ9102’, were surface-sterilized in 15% (*v*/*v*) H_2_O_2_ for 30 min, thoroughly rinsed with deionized water, soaked for 24 h, and then sown in moist, well-washed quartz sand [32]. Seven-day-old seedlings at the first trifoliate stage were transferred into 3 L plastic pots containing aerated nutrient solution. To minimize potential interference from seed-stored Fe reserves on subsequent growth and physiological responses, cotyledons were excised at the time of transplantation [33]. The basal nutrient solution had the following composition (in mmol·L^−1^): K_2_SO_4_ 0.75, Ca(NO_3_)_2_ 2.0, KH_2_PO_4_ 0.25, MgSO_4_ 0.65, H_3_BO_3_ 10 × 10^−3^, MnSO_4_·H_2_O 1 × 10^−3^, ZnSO_4_·7H_2_O 1 × 10^−3^, CuSO_4_·5H_2_O 0.5 × 10^−3^, and (NH_4_)Mo_7_O_24_·4H_2_O 0.05 × 10^−3^. The pH was adjusted to 6.30 ± 0.02 using NaOH or HCl.

Plants were subjected to two Fe treatments: Fe-deficient (0 µmol·L^−1^ Fe, −Fe) and Fe-sufficient (40 µmol·L^−1^ Fe supplied as FeSO_4_·7H_2_O, +Fe). The nutrient solution was completely renewed every three days. Plants were grown in a controlled greenhouse with a 13 h photoperiod, a light intensity of 500–600 µmol photons·m^−2^·s^−1^, and a day/night temperature regime of 25 ± 2 °C. Pots were re-randomized daily. The experiment followed a two-factor (cultivar × Fe treatment) completely randomized design with five replications (pots) per treatment combination, each containing six plants.

#### 2.1.2. Plant Tissue Analysis

At 35 days after treatment (DAT), plants were collected and separated into leaves, stems, and roots. Samples were rinsed in deionized water, oven-dried to constant weight at 70 °C, and ground into a fine powder. For total Fe determination, approximately 0.2 g of dried tissue was digested in a 5:1 (*v*/*v*) mixture of concentrated HNO_3_ and HClO_4_. Fe concentration in the digests was measured using a flame atomic absorption spectrophotometer (WFX-120C, Beijing Rayleigh Analytical Instrument Corp., Beijing, China) [34].

#### 2.1.3. Determination of Active Fe and Chl

The concentration of active (HCl-extractable) Fe in young leaves was determined at 21 DAT. Fresh leaf tissue (2.0 g) was shaken in 20 mL of 1 mol·L^−1^ HCl for 5 h. The Fe content in the resulting filtrate was analyzed by atomic absorption spectrometry as above [35].

Leaf Chl concentration was assessed nondestructively at 20 DAT using a SPAD-502 chlorophyll meter (Konica Minolta, Tokyo, Japan). For each treatment, measurements were taken at five locations on the first fully expanded leaf, with ten replicate leaves sampled.

#### 2.1.4. Pn and Chl Fluorescence Measurements

The Pn of the first fully expanded leaf was recorded at 21 DAT between 09:00 and 11:00 using a LI-6400XT portable photosynthesis system (LI-COR Biosciences, Lincoln, NE, USA). During measurements, the leaf chamber was set to maintain a photosynthetic photon flux density of 1800 μmol·m^−2^·s^−1^, a leaf temperature of 30 °C, and a CO_2_ concentration of 400 µmol·mol^−1^.

On the same leaves following 20 min of dark adaptation, Chl fluorescence parameters were determined with a portable PEA fluorometer (Hansatech Instruments Ltd., Norfolk, UK). After the application of a saturating light pulse (>3000 µmol·m^−2^·s^−1^), minimal fluorescence (F_0_) and maximum fluorescence (F_m_) were recorded. The maximum quantum efficiency of photosystem II (PSII) was derived as F_v_/F_m_ = (F_m_ − F_0_)/F_m_ [36].

#### 2.1.5. Rhizosphere Acidification and Root FCR Activity

Rhizosphere acidification capacity was evaluated by recording the pH of the nutrient solution from 6 to 9 DAT. The solution was not replaced during this 72 h period; only deionized water was replenished to offset losses from evapotranspiration. Solution pH was monitored using a calibrated pH meter (IQ150, IQ Scientific Instruments, San Diego, CA, USA). The lack of a plant-free control treatment is a limitation of this study, as the observed changes in pH may have resulted from both plant-mediated processes and abiotic factors.

Root FCR activity was determined at 21 DAT following established protocols [33,37]. Intact root systems were washed, immersed in saturated CaSO_4_ for 5 min, and then transferred in triangular flasks wrapped with black plastic. Each triangular flask contained 40 mL of reaction solution containing (mmol·L^−1^) K_2_SO_4_ 0.75, Ca(NO_3_)_2_ 2.0, KH_2_PO_4_ 0.25, MgSO_4_ 0.65, H_3_BO_3_ 10 × 10^−3^, MnSO_4_·H_2_O 1 × 10^−3^, ZnSO_4_·7H_2_O 1 × 10^−3^, CuSO_4_·5H_2_O 0.5 × 10^−3^, and (NH_4_)Mo_7_O_24_·4H_2_O 0.05 × 10^−3^. The initial pH of reaction solution was at 5.0. An assay was conducted under environmental conditions matching those of plant growth in the greenhouse. Following a 2 h reaction period, the concentration of the Fe^2+^–dipyridyl complex was measured at absorbance under 523 nm using a spectrophotometer (Specord 200 Plus, Analytik Jena AG, Jena, Germany). The nutrient solution contained 0.1 mmol·L^−1^ Fe^3+^-EDTA and 0.4 mmol·L^−1^ 2, 2-bipyridyl as the blank. The Fe reduction capacity of the roots was assessed by the formation of the Fe^2+^–dipyridyl complex.

#### 2.1.6. RT-PCR of AhIRT1 and AhFRO1

Total RNA in the roots at 21 DAT was extracted with a Trizol reagent (TaKaRa Biomedicals, Shiga, Japan). First-strand cDNA was synthesized from 1 µg of total RNA in a 10 µL reaction volume containing an anchored oligo (dT) primer and 200 U of Moloney murine leukemia virus (M-MLV) reverse transcriptase (TaKaRa Biomedicals, Tokyo, Japan), following the supplier’s instructions.

Quantitative real-time PCR (qRT-PCR) was conducted on the ABI 7500 system (Biosystems, Foster City, CA, USA) using the SYBR Premix Ex Taq kit (TaKaRa Biomedicals, Tokyo, Japan) based on the manufacturer’s protocol. Gene-specific primers (Table 1) were used to amplify *AhFRO1* (primers FROP1/FROP2) and *AhIRT1* (primers IRTP1/IRTP2). The peanut *Actin* gene served as an internal reference for data normalization [38,39]. The thermal cycling protocol consisted of an initial step at 94 °C for 5 min, followed by 30 cycles of denaturation at 94 °C for 30 s, annealing at 52 °C for 30 s, and extension at 72 °C for 30 s. Transcript levels were analyzed using the comparative Ct method, with three technical replicates for each sample.

### 2.2. Experiment 2: Agronomic Response of Soil-Grown Peanut to Fe Fertilization

#### 2.2.1. Soil-Grown Panut and Treatment

A pot experiment was conducted in a greenhouse. Fluvo-aquic soil with sandy texture was sampled from Hebei province in Northern China. The soil pH (soil–H_2_O as 1:2.5) was determined as 7.88, organic matter 16.6 g·kg^−1^, total nitrogen 0.773 g·kg^−1^, Olsen-P 55.69 mg·kg^−1^, and NH_4_AC-K 137.50 mg·kg^−1^. The concentration of soil DTPA-Fe prior to cropping was 6.96 mg·kg^−1^. The soil saturated water capacity was 28% (*w*/*w*). Air-dried soil that passed through a 2 mm sieve was weighed (4.7 kg) and placed into polyethylene pots (15.0 cm diameter, 23.5 cm height).

Seeds of similar size were surface-sterilized in 10% (*v*/*v*) H_2_O_2_ for 30 min, thoroughly rinsed with deionized water, and germinated on filter paper at 25 °C for 48 h. Initially, seven uniformly germinated seeds were sown per pot and thinned to five plants after emergences. To minimize the influence of seed-stored Fe on growth, cotyledons were removed from the seedlings. Deionized water was irrigated to soil. Soil moisture was maintained at 60% of saturated water capacity by daily weight-based replenishment with deionized water; watering was triggered when the soil moisture decreased by 0.01–0.02 g·g^−1^. The bulk density of the soil in pots was approximately 1.70 g·cm^−3^. Before plant sowing, N, P, and K fertilizers as potassium nitrate and calcium dihydrosulfate were mixed with soil and applied at the rates of 0.045 g·kg^−1^ N, 0.15 g·kg^−1^ P_2_O_5_, and 0.15 g·kg^−1^ K_2_O. Plants were treated with 0 (Fe) and 5 mg·kg^−1^ Fe (+Fe) as ferrous sulfate. Each treatment was repeated three times, and pots were arranged randomly.

#### 2.2.2. Plant Growth and Analysis

The soil experiment comprised two consecutive cropping cycles. The first crop was terminated at 50 DAT, at which time plant samples were collected. After sample collection, soil from each pot was carefully removed, visible root residues from the first crop were manually removed, and the soil was homogenized before being repotted for the second crop. The second crop was terminated at 42 DAT.

At the end of each cycle, shoots were separated, rinsed, dried (105 °C for 30 min, then 65–70 °C to constant weight), and weighed for biomass determination. Dried shoot samples were ground, digested, and analyzed for total Fe concentration, as described in Section 2.1.2. Active Fe in young leaves (sampled at 30 DAT for each cycle) and leaf Chl content (SPAD value, measured at 35 DAT for each cycle) were determined using the methods outlined in Section 2.1.3.

#### 2.2.3. Statistical Analysis

Data were analyzed using a one-way analysis of variance (ANOVA). Differences between treatments were assessed with Duncan’s multiple range test at the 5% significance level.

## 3. Results

### 3.1. Biomass

**Figure 1:**
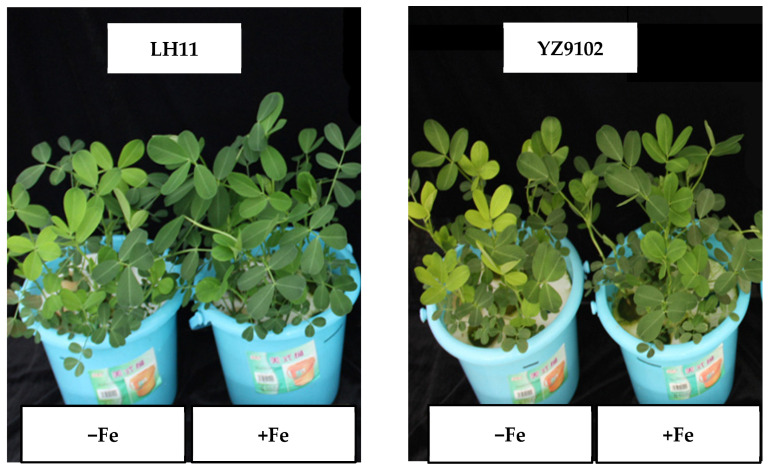
LH11 and YZ9102 cultured in nutrient solution with Fe treatments: 0 µmol·L^−1^ (−Fe) and 40 µmol·L^−1^ (+Fe). Under −Fe treatment, LH11 young leaves showed light chlorosis symptoms, while YZ9102 appeared to have obvious Fe deficiency chlorosis.

In the nutrition solution experiment, the Fe deficiency symptom of chlorosis (Figure 1) was shown in the young leaves of YZ9102 and LH11 at 5 DAT and 7 DAT, respectively. YZ9102 had lower biomass than that of LH11. The root dry weight of the two peanut cultivars did not change under Fe deficiency, while the root biomass of YZ9102 was less than that of LH11. The root–shoot ratio of YZ9102 was significantly higher than that of LH11 under limited Fe supply (Table 2).

In the first growth period of the soil experiment, peanut plants without Fe fertilizer did not show Fe deficiency symptoms. Fe application to soil did not affect the shoot dry weight of peanut. In the second growth period, the peanut YZ9102 without Fe fertilizer showed chlorosis at 14 DAT, while LH11 did not. The shoot biomass of YZ9102 decreased when no Fe was added to soil (Table 3).

### 3.2. Total Fe and Active Fe Concentrations in Plants

Regardless of Fe application, there was no significant difference in Fe concentration in roots between the two peanut varieties (Table 2). Fe application did not affect Fe concentration in roots (Table 2), while it increased Fe concentration in shoots (Table 2 and Table 3).

Fe application increased the active Fe concentration in the young leaves of both peanut varieties. Without Fe addition, the concentration of active Fe in the young leaves of LH11 was slightly higher than that of YZ9102 (Table 2). However, the concentration of active Fe in the young leaves of peanut plants showed no significant difference between Fe treatments in soil (Table 3).

### 3.3. Photosynthetic Parameters

The SPAD value, an index of total Chl, of young leaves did not change with Fe fertilization in the first growth period when grown in soil. In the second period, the SPAD value of YZ9102 leaves without Fe application declined significantly, while LH11 showed no change (Table 3).

Under limited Fe supply in nutrient solution, the SPAD value and Pn of YZ9102 were significantly lower than those of LH11. Fe application significantly increased the leaf SPAD value and Pn of LH11 and YZ9102 (Table 4). Compared with LH11, the F_v_/F_m_ of YZ9102 was significantly lower under the condition of no Fe addition, indicating that the PSII maximum photochemical efficiency of YZ9102 was inhibited by Fe deficiency. Fe application slightly increased the F_v_/F_m_ of peanut LH11, while it significantly enhanced the F_v_/F_m_ of peanut YZ9102 (Table 4).

### 3.4. FCR Activity in Roots

Without Fe application in nutrient solution, the activities of FCR in the roots of LH11 and YZ9102 peanuts were 2.1 and 1.3 times higher than those with Fe treatment, respectively. Fe application significantly reduced the activity of root FCR. Under Fe deficiency stress, the root FCR activity of LH11 peanut was significantly higher than that of YZ9102 (Table 4).

### 3.5. pH of Growth Medium

**Figure 2:**
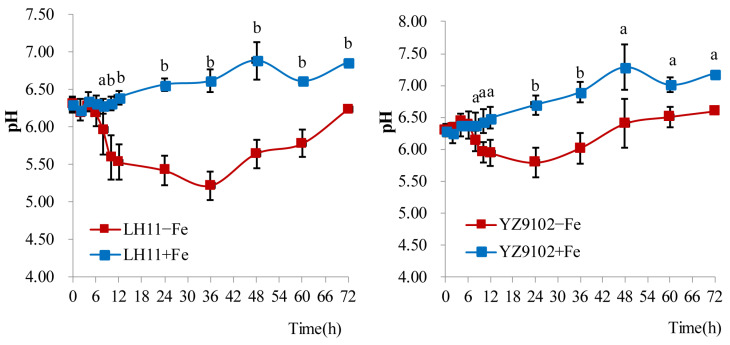
The change in pH in nutrient solution for growing peanut cultivars LH11 (**left**) and YZ9102 (**right**) with Fe treatments: 0 µmol·L^−1^ (−Fe) and 40 µmol·L^−1^ (+Fe). Error bars indicate the standard deviation (SD) (*n* = 4). Different letters in the figure indicate statistical significance: ‘a’ for *p* < 0.05 and ‘b’ for *p* < 0.01.

Under no Fe supply, the pH value of nutrient solution for LH11 was the lowest at 36 h, which was 1.08 units lower than the initial pH value, and then rose slightly to reach the initial pH level at 72 h. The pH value of growth medium for YZ9102 was the lowest at 24 h, which was 0.50 units lower than the initial pH value, and it then increased to 0.30 units higher than the initial pH at 72 h. Compared with YZ9102, the pH value of peanut LH11 growth medium decreased significantly without Fe addition (Figure 2). At the late stage, the increase in the pH of the nutrient solution of both cultivars was related to nitrate being the nitrogen source for growing plants [6].

### 3.6. AhIRT1 and AhFRO1 Expression

**Figure 3:**
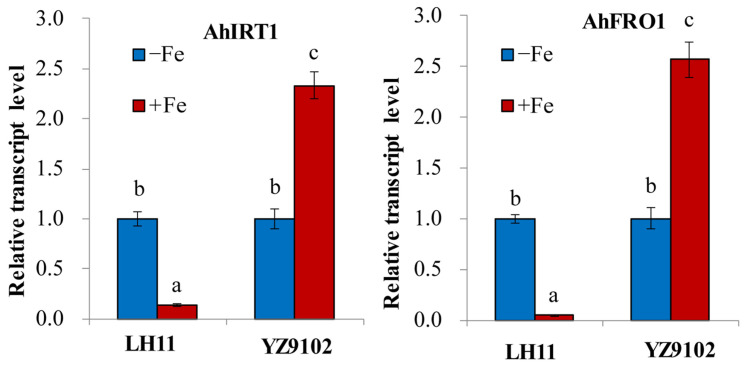
The accumulation of *AhIRT1* and *AhFRO1* transcripts in peanut roots was examined through real-time PCR under Fe-deficient conditions. Expression levels were calculated relative to *Actin*, with the control set as 1.0. Data represent means ± SD from three biological replicates. Significant differences (*p* < 0.05) between treatments are marked with distinct letters (*n* = 3).

*AhFRO1* encoding FCR and *AhIRT1* encoding IRT1 are key genes for Fe(II) acquisition in root cells in peanut plants. Under Fe-deficient conditions, the transcript level of *AhIRT1* and *AhIFRO1* in the roots of LH11 increased, while the transcript level of *AhIRT1* and *AhIFRO1* in the roots of YZ9102 decreased (Figure 3).

## 4. Discussion

Peanut is a crop sensitive to Fe deficiency, which adversely influences plant growth and metabolism. There are large variations in Fe deficiency tolerance among peanut cultivars [3,4,35]. In earlier studies, Pattanashetti et al. [40] first identified the QTLs associated with resistance to Fe deficiency chlorosis using a recombinant inbred line population (TAG 24 × ICGV 86031) over two consecutive years and three growth stages. Thirty-two QTLs were detected for visual chlorosis rating and SPAD Chl meter reading, which showed 3.9–31.8% phenotypic variance explained (PVE) and 3.8–11% PVE, respectively. Our study showed that Fe application did not affect Fe concentration in peanut roots, while it increased Fe concentration in shoots (Table 2 and Table 3). Peanut cultivars YZ9102 and LH11 showed different responses to Fe deficiency stress. Fe deficiency symptoms of chlorosis in young leaves appeared earlier in YZ9102 than LH11 when grown in the nutrient solution without Fe addition (Figure 1). In the second growth period of the pot experiment, the peanut YZ9102 grown in soil without Fe fertilizer appeared to show chlorosis, while LH11 did not show Fe deficiency symptoms.

During the first growth period, Fe application did not significantly influence the shoot dry weight of peanut plants. However, the shoot biomass of YZ9102 was reduced when grown without Fe fertilizer (Table 3). The decrease in the shoot biomass of YZ9102 was more serious than that of LH11 (Table 2 and Table 3). No Fe addition resulted in a decline in the total Fe concentration in shoots and the concentration of active Fe in the young leaves of both peanut cultivars YZ9102 and LH11 grown in nutrient solution. Moreover, LH11 shoots had higher concentrations of total Fe and active Fe than YZ9102. The root biomass of YZ9102 was lower than that of LH11. The stimulation of root growth under Fe-deficient conditions may represent an Fe efficiency mechanism, potentially enhancing Fe mobilization and uptake from the medium [41].

Fe deficiency impairs photosynthesis in plants [6,12], a response linked to reduced photosynthetic pigment content [42]. The present study showed that decreases in Pn and the SPAD value as the index of the Chl content were greater in YZ9102 than in LH11 under Fe deficiency stress (Table 3 and Table 4). Fe plays a critical functional role within photosynthetic complexes, where it participates directly in electron transfer. The maximum efficiency of PSII in plants can be estimated using the F_v_/F_m_ value determined by fluorescence analysis [43]. Su et al. [3] found that Fe deficiency significantly decreased F_v_/F_m_ for most peanut cultivars except ‘Baisha 1016’ and ‘Zhenghong 3’. Our observation showed that the F_v_/F_m_ of YZ9102 was lower under Fe deficiency stress in comparison to LH11, indicating that the inhibition of the PSII maximum photochemical efficiency under Fe deficiency was greater in YZ9102 than LH11 (Table 4).

Under low-Fe-availability conditions, plants have developed two principal Fe uptake strategies: Strategy I employed by nongraminaceous monocots and dicots and Strategy II specific to graminaceous monocots [6,15]. The strategies used by nongraminaceous monocots and dicots plants include the following: (i) rhizosphere acidification via proton extrusion [20], (ii) the reduction of insoluble Fe(III) to soluble Fe(II) by root surface FCR activity [21], and (iii) the transmembrane transport of Fe(II) into root epidermal cells by the IRT1 transporter [23]. As a Strategy I plant, peanuts possess orthologous genes involved in Fe acquisition, including *AhIRT1* [39], *AhNramp1* [44], and *AhFRO1* [38]. Research indicates that Fe deficiency induced the expression of transporters like *Nramp5* and *IRT1* in peanut roots, a response also linked to increased Cd uptake [26]. Nevertheless, the molecular basis for varietal differences in Fe deficiency tolerance remains poorly characterized. Ding et al. [38] found that the higher FCR activity and higher transcript levels of *AhFRO1* occurred in peanut–maize intercropping than monocropped peanut. The optimal pH for FCR activity in Fe-deficient peanuts is approximately 5.0, with significant inhibition under alkaline conditions [45]. Furthermore, varietal variation exists: peanut cultivar Silihong showed a greater capability for Fe uptake and translocation compared to cultivar Fenghua 1, correlating with the higher expression level of genes such as *AHA4, FRO2, IRT1*, and *Nramp5* [46]. In the present research, we observed that the decrease in pH in the growth medium of peanut LH11 without Fe addition was greater than that of YZ9102 (Figure 2). The root FCR activity of LH11 peanut was also significantly higher than that of YZ9102 (Table 4). Moreover, the transcript levels of *AhIRT1* and *AhIFRO1* in the roots of LH11 increased under the Fe-deficient condition, while they decreased in the roots of YZ9102 (Figure 3). It is suggested that peanut cultivar LH11 seedlings have a high Fe(II) uptake capacity together with high FCR activity, which may contribute to their greater ability to alleviate Fe deficiency chlorosis compared with YZ9102. Thus, LH11 may be considered an Fe-deficiency-tolerant cultivar, whereas YZ9102 appears to be more sensitive to Fe deficiency (Figure 3). Interestingly, this tolerance contrast appears element-specific. Gao et al. [32] found that peanut cultivar YZ9102 had greater tolerance to calcium (Ca) deficiency than LH11, attributed to its higher Ca uptake and translocation efficiency. The relationship between peanut YZ9102 and LH11 plant adaptation to Fe deficiency and Ca deficiency is unclear, which should be further studied.

## 5. Conclusions

This study demonstrates that the peanut cultivar LH11 exhibits significantly greater tolerance to Fe deficiency compared to the cultivar YZ9102. The enhanced tolerance of LH11 is attributed to a coordinated suite of physiological and molecular adaptations under Fe-deficient conditions, including the superior maintenance of photosynthetic function, increased root biomass, more efficient rhizosphere acidification, and significantly higher root FCR activity. Crucially, these physiological advantages are supported by the up-regulation of key Fe-acquisition genes, *AhIRT1* and *AhIFRO1*, in the roots of LH11, thereby facilitating improved Fe mobilization and uptake. Therefore, LH11 represents a valuable Fe-deficiency-tolerant germplasm resource. Nevertheless, the specific role of plant-induced processes in rhizosphere acidification should be further validated in controlled setups that include unplanted treatments. The contrasting adaptation strategies of YZ9102 and LH11 to Fe and calcium deficiency, as noted in previous studies, highlight the complexity of nutrient stress interactions and warrant further investigation.

## Figures and Tables

**Table 1:** Primers used for detecting the expression levels of *AhFRO1* and *AhIRT1* in peanut roots.

Primer Name	Sequence (5′-3′)
FROP1	ATGATCTTCTCCATCTTCCA
FROP2	TGCCACTTCCTCCACTAACC
IRTP1	AAGATGGAGACACACAACTCGTGC
IRTP2	GCCAAGACCAATGCCTTCAAACAT
ACTIN1	GCTACCAGATGGACAGGTTATCAC
ACTIN2	ACCACCACTCAAGACAATGTTACC

**Table 2:** Plant dry matter weights, total Fe concentration, and active Fe concentration in young leaves of two peanut cultivars grown in nutrient solution with Fe treatments: 0 µmol·L^−1^ (−Fe) and 40 µmol·L^−1^ (+Fe).

Cultivars	Fe Treatments	Dry Matter Weights (g per Plant)		Shoot/Root Ratio	Active Fe Content (mg·kg^−1^ Fresh Weight)	Fe Concentration (mg·kg^−1^ Dry Weight)	
		Roots	Shoots			Roots	Shoots
LH11	−Fe	0.196 ± 0.045 ab	1.121 ± 0.209 b	0.17 ± 0.01 a	7.06 ± 0.54 b	200.60 ± 17.00 ab	350.92 ± 36.09 b
	+Fe	0.208 ± 0.030 b	1.370 ± 0.174 c	0.15 ± 0.01 a	10.37 ± 0.91 c	218.41 ± 7.46 b	1627.88 ± 47.11 d
YZ9102	−Fe	0.153 ± 0.022 a	0.710 ± 0.128 a	0.22 ± 0.02 b	5.79 ± 0.52 a	180.94 ± 24.45 a	175.18 ± 15.18 a
	+Fe	0.145 ± 0.016 a	0.846 ± 0.096 a	0.17 ± 0.01 a	10.41 ± 1.84 c	204.33 ± 5.22 ab	1433.82 ± 93.18 c

Notes: Data are mean ± SD (*n* = 4). Different small letters after data in same column indicate significant difference at *p* < 0.05 level.

**Table 3:** Shoot biomass, total Fe concentration, active Fe concentration, and SPAD values in young leaves of two peanut cultivars grown in soil without Fe and with Fe fertilization.

Cultivars	Fe Treatments	The First Growth Period				The Second Growth Period			
		Shoot Biomass (g per Plant)	Fe Concentration in Shoot (mg·kg^−1^ Dry Weight)	Active Fe Content (mg·kg^−1^ Fresh Weight)	SPAD Value	Shoot Biomass (g per Plant)	Fe Concentration in Shoot (mg·kg^−1^ Dry Weight)	Active Fe Content (mg·kg^−1^ Fresh Weight)	SPAD Value
LH11	−Fe	1.464 ± 0.129 b	200.20 ± 17.83 a	15.02 ± 0.39 a	48.23 ± 2.10 b	0.989 ± 0.124 c	199.46 ± 15.16 a	11.25 ± 1.66 a	35.30 ± 1.81 b
	+Fe	1.355 ± 0.055 b	319.68 ± 62.87 ab	17.05 ± 1.22 a	48.07 ±1.95 ab	1.044 ± 0.078 c	199.64 ± 21.92 a	10.89 ± 0.67 a	36.27 ± 1.34 b
YZ9102	−Fe	1.096 ± 0.125 a	270.93 ±17.99 a	16.80 ± 2.08 a	45.03 ± 0.95 a	0.629 ± 0.048 a	238.60 ± 22.29 a	12.70 ± 0.31 a	29.73 ± 1.46 a
	+Fe	1.132 ± 0.133 a	424.83 ± 108.00 b	15.24 ± 0.81 a	45.27 ± 0.72 ab	0.817 ± 0.085 b	215.30 ± 17.90 a	11.28 ± 1.91 a	34.30 ± 0.62 b

Notes: Data are mean ± SD (*n* = 3). Different small letters after data in same column indicate significant difference at *p* < 0.05 level.

**Table 4:** The SPAD value, Pn, and F_v_/F_m_ of leaves and the activity of FCR in the roots of two peanut cultivars grown in nutrient solution with Fe treatments: 0 µmol·L^−1^ (−Fe) and 40 µmol·L^−1^ (+Fe).

Cultivars	Fe Treatments	SPAD Value	Pn (μmol·m^−2^·s^−1^)	F_v_/F_m_	Activity of FCR in Roots (μmol·g^−1^ Fresh Weight·h^−1^)
LH11	−Fe	35.15 ± 0.75 b	16.2 ± 1.6 b	0.810 ± 0.012 b	3.15 ± 0.92 b
	+Fe	40.53 ± 0.80 c	17.8 ± 0.9 b	0.831 ± 0.007 b	1.48 ± 0.43 a
YZ9102	−Fe	28.28 ± 2.09 a	2.2 ± 0.4 a	0.753 ± 0.006 a	2.05 ± 0.30 ab
	+Fe	40.50 ± 0.94 c	15.3 ± 0.7 b	0.830 ± 0.012 b	1.61 ± 0.17 a

Notes: Data are mean ± SD (*n* = 4 except for Pn with *n* = 3). Different small letters after data in same column indicate significant difference at *p* < 0.05 level.

## Data Availability

The original contributions presented in this study are included in the article. Further inquiries can be directed to the corresponding author.
